# Neuronal Correlates of Informational and Energetic Masking in the Human Brain in a Multi-Talker Situation

**DOI:** 10.3389/fpsyg.2019.00786

**Published:** 2019-04-09

**Authors:** Orsolya Szalárdy, Brigitta Tóth, Dávid Farkas, Erika György, István Winkler

**Affiliations:** ^1^Institute of Cognitive Neuroscience and Psychology, Research Centre for Natural Sciences, Hungarian Academy of Sciences, Budapest, Hungary; ^2^Institute of Behavioural Sciences, Faculty of Medicine, Semmelweis University, Budapest, Hungary

**Keywords:** perceptual masking, EEG functional networks, neural oscillations, speech processing, selective attention

## Abstract

Human listeners can follow the voice of one speaker while several others are talking at the same time. This process requires segregating the speech streams from each other and continuously directing attention to the target stream. We investigated the functional brain networks underlying this ability. Two speech streams were presented simultaneously to participants, who followed one of them and detected targets within it (target stream). The loudness of the distractor speech stream varied on five levels: moderately softer, slightly softer, equal, slightly louder, or moderately louder than the attended. Performance measures showed that the most demanding task was the moderately softer distractors condition, which indicates that a softer distractor speech may receive more covert attention than louder distractors and, therefore, they require more cognitive resources. EEG-based measurement of functional connectivity between various brain regions revealed frequency-band specific networks: (1) energetic masking (comparing the louder distractor conditions with the equal loudness condition) was predominantly associated with stronger connectivity between the frontal and temporal regions at the lower alpha (8–10 Hz) and gamma (30–70 Hz) bands; (2) informational masking (comparing the softer distractor conditions with the equal loudness condition) was associated with a distributed network between parietal, frontal, and temporal regions at the theta (4–8 Hz) and beta (13–30 Hz) bands. These results suggest the presence of distinct cognitive and neural processes for solving the interference from energetic vs. informational masking.

## Introduction

When two different sound streams are presented at the same time, the auditory system is able to separate them even though they may be interleaved and/or overlapped in time and frequency (auditory scene analysis, see Bregman, 1990). However, each stream will still interfere with the processing of others (termed auditory masking). Two forms of masking may occur while listening to two competing speech streams: energetic and informational masking (Freyman et al., 1999; Brungart et al., 2001; Arbogast et al., 2002). Whereas energetic masking influences the separation of the speech streams due to the higher energy of the masker, informational masking denotes the interference between similar stimuli. Energetic masking affects the early part of sound processing in the brain, whereas information masking probably affects later processes, and so it may occur even after stream segregation. While behavioral studies extensively investigated these masking effects on speech perception (Arbogast et al., 2002; Freyman et al., 2004; Ihlefeld and Shinn-Cunningham, 2008) the underlying neural processes are poorly understood. In the current study, the cognitive and underlying neural mechanism for informational and energetic masking were assessed in dichotic listening conditions by combining the measurements of behavioral psychophysics and cortical electrophysiology (event-related potential [ERP] and functional connectivity [FC] analysis).

Previous studies comparing energetic and informational masking showed that these two masking processes lead to different patterns of errors in task performance. Target detection is reduced when a speech segment is masked by high-level noise (energetic masking), whereas allocation problems are found when a speech stream is masked by another one(informational masking, Darwin, 2008). For example, in tasks requiring listeners to detect words in one of two concurrent speech streams, informational masking increases the number of words reported from the masker streams (Kidd et al., 2005; Wightman and Kistler, 2005). In contrast, when energetic masking is dominant, errors of omission occur more frequently. This picture has been further elaborated by the study of Ihlefeld and Shinn-Cunningham (2008). They used a selective listening task with two speech streams presented concurrently. One of the streams served as the target, the other as the distractor, and the target-to-masker energy ratio was varied. The participants’ task was to report the presence of the two target words after the end of the sentences. Participants produced more correct and fewer missed responses as energetic masking decreased (the target became louder relative to the masker). Unexpectedly, masker errors (when participants reported words from the masker stream) were higher when the target-to-masker energy ratio was 1 or higher, and informational masking started to dominate over energetic masking. The highest number of masker errors occurred when the target-to-masker energy ratio was 1 (i.e., the loudness of the two speech streams was equal). This observation indicates that informational and energetic masking involve different cognitive and thus different underlying neural processes.

Three important mechanisms may contribute to word detection in such selective listening tasks. The first one is short-term segmentation when the acoustic mixture is segregated into streams based on the spectro-temporal structure of sound sources (see, e.g., Carlyon, 2004). When energetic masking is strong, this process becomes less accurate and may require additional resources. The second mechanism is linking speech elements across time (i.e., when the shorter segments are grouped into continuous streams). This mechanism can be impaired in case of high perceptual similarity between the target and masker. The third mechanism is the attentional selection. Even if the streams are properly formed the listener must select the correct one (Desimone and Duncan, 1995). Therefore, while early perceptual processes can (partly) compensate for the effects of energetic masking, later selection processes may be required to alleviate the effects informational masking (Ihlefeld and Shinn-Cunningham, 2008).

The impact of selective attention on target speech processing has been investigated in several electrophysiological (EEG) experiments (see, e.g., Hansen et al., 1983; Wild-Wall and Falkenstein, 2010). In general, two ERP components elicited by auditory target events (including speech stimuli) are strongly modulated by selective attention: the N2b (Näätänen et al., 1982; Pritchard et al., 1991) and the P3b (for reviews, see Sutton et al., 1965; Donchin and Coles, 1988; Polich, 2007). The N2b is a negative waveform with typical central distribution, peaking at around 200 ms from stimulus onset. It has been typically associated with stimulus classification (Ritter et al., 1979). The P3b often follows the N2b. It appears at around 300–400 ms from the target onset with positive polarity and parietally dominant scalp distribution. P3b has been interpreted as reflecting context updating (Donchin and Coles, 1988) or closure of the target detection cycle (Verleger, 1988) or as a sign of interaction between working memory and attentional processes (Polich and Herbst, 2000). Both components have been found for target speech stimuli in a previous study using continuous speech streams (Szalárdy et al., 2018). In another ERP study which investigated informational and energetic masking on speech processing (Zhang et al., 2014) the amplitude of the N1/P2 component was smaller in the informational than in the energetic masking conditions for a target syllable. However, they did not analyze the N2 and P3 components which are typical for target events and may show modulation by the type of the masking effect.

Whereas ERPs provides information about the masking effects on event processing (such as target detection), measuring FC changes indexes sustained mental processes. FC (measured as neural oscillatory phase synchronization in EEG; Stam and van Straaten, 2012) describes how the different brain areas are configured into networks during performing the given task, where the brain regions are functionally connected through oscillatory activity. The analysis of FC can also potentially reveal interactions between different brain networks, such as those supporting attentional control and speech processing functions (Najafi et al., 2016) as oscillatory synchrony between cortical regions is assumed to facilitate neuronal communication (Fell and Axmacher, 2011). Up to now, only a few EEG studies investigated speech processing in dichotic listening tasks using FC measured from the EEG (Brancucci et al., 2008; Tóth et al., 2019). Brancucci et al. (2008) found that spectral coherence of the high-alpha band rhythms is increased between the left auditory cortex and Wernicke’s area with large spectral overlap between the presented syllables (for instance /da/-/ba/) compared to lower spectral overlap (/da/-/ka/). This result may indicate that solving the problem of energetic masking is associated with stronger alpha synchronization. In contrast to energetic masking, solving the problem of informational masking may be associated with brain networks underlying selective attention. In a situation when listeners were presented with two speech streams of equal loudness, Tóth et al. (2019) found strong activity in networks previously associated with selective attention (e.g., a fronto-parietal network in the alpha band; see below) when listeners were instructed to listen to one (focused attention) as opposed to two streams (divided attention).

It is commonly observed that EEG low-frequency phase synchronization increases between the frontal and parietal brain regions in tasks requiring attentional orientation (Brazdil et al., 2013; Daitch et al., 2013; Dombrowe and Hilgetag, 2014). Attention has been shown to modulate brain oscillations in the delta (0.5–4 Hz) and theta (4–8 Hz) frequency bands, which have been associated with information selection (Schroeder and Lakatos, 2009; Herrmann et al., 2016). Several studies described that the posterior medial frontal cortex (pMFC) and the lateral prefrontal cortex (LPFC) are crucial hubs of this network coordinating their activity through theta-band phase synchronization: the LPFC transmits excitatory or inhibitory signals to regions involved in representing the input (MacDonald et al., 2000; Ridderinkhof et al., 2004).

Alpha-band (8–13 Hz) activity is also often enhanced in selective attention tasks. Alpha oscillation has been assumed to contribute sustained attention, albeit through different processes than the aforementioned delta/theta oscillations (Makeig and Jung, 1995; Braboszcz and Delorme, 2011). Enhanced alpha-band activity is associated with the disengagement of task-irrelevant cortical areas (Cooper et al., 2003; Klimesch et al., 2007) and thus, a marker of inhibition. With respect to speech processing, low alpha power characterize brain regions involved in speech processing when speech is presented in a quiet environment. Adding a masker then results in higher alpha activity in line with the assumed inhibitory role of the alpha oscillations (Strauss et al., 2014). Further, alpha power was increased in auditory cortical areas contralateral to the side from which the masker was presented and decreased contralateral to the side of the target speech, indicating that auditory cortex can selectively amplify the neural correlates of the attended speech signal (Kerlin et al., 2010). Therefore the impact of alpha power and synchrony seems twofold: global alpha enhancement (particularly involving the fronto-parietal network) possibly contributes to the maintenance of sustained attention whereas enhanced alpha activity over the task-irrelevant cortical regions may reflect the inhibitory activity triggered by the to-be-suppressed stimuli; the latter process is also driven by the frontal control regions (Liu et al., 2016).

Whereas communication through slower brain oscillations can connect distant brain areas and distributed networks, faster oscillations are involved in mediating more local processes affecting sensory brain areas. Gamma (>30 Hz) oscillation over the sensory cortices is often linked with enhanced attention to the sensory events both in the visual (Reinhart et al., 2011; Akimoto et al., 2013) and in the auditory domain (Ahveninen et al., 2013; Potes et al., 2014). Therefore local gamma oscillations presumably promote task-related sensory processes in the brain.

The current study investigated neural mechanisms of energetic and informational masking during selective listening to test whether these two types of masking recruit different neural mechanisms in the human brain. Two concurrent continuous speech streams were presented to listeners who were instructed to track the contents of one of them and detected targets within that stream (attended speech) while ignoring the other (unattended/distractor speech). The loudness of the distractor stream was varied on five levels: moderately softer, slightly softer, equal, slightly louder, or moderately louder than the target stream. When the distractor is softer than the attended stream, based on previous work (Ihlefeld and Shinn-Cunningham, 2008), we expected informational masking to be stronger than energetic masking and vice versa, stronger energetic masking when the distractor stream is louder. The target detection task should be more sensitive to local masking effects (impairing short-term segmentation and possibly target selection), while the content tracking task would be more affected by more global masking effects (impairing the linking speech of elements across time and maintaining focus on the target stream). Both types of masking exert local effects, but only information masking is expected to produce more global effects (i.e., while either type of masking could make the detection of a single word more difficult, only information masking could lead to breaking the continuity of the target stream and confusion between the concurrent streams). We assumed that solving the selective listening task when energetic masking is dominant may require selective enhancement of the task-relevant sensory input. Therefore, we hypothesize that energetic masking will elicit stronger local connectivity in temporal and parietal areas in the gamma band (enhancement/difficulty of auditory sensory processes) together with stronger large scale fronto-parietal connectivity in the alpha band (increasing suppression of the non-target sounds). In contrast, solving the informational masking problem may predominantly recruit processes involved in higher-level attentional selection. Therefore we hypothesize that informational masking will elicit stronger large scale FC within the attentional control network linked to the frontal hub in the delta (0.5–4 Hz) and theta (4–8 Hz) bands.

## Materials and Methods

### Participants

Twenty-eight young healthy adults (20 female, mean age: 22 years, SD: 2,867, 24 right-handed), all native Hungarian speakers participated in the experiment. Participants had normal hearing measured by pure-tone audiometry between the frequencies ranging from 250 to 4 kHz (<25 dB, separately for the two ears and <10 dB difference between ears). All participants signed an informed consent form and received modest financial compensation for their participation. Based on self-reports, none of them had a history of psychiatric or neurological disorders. The experiment was conducted in full accordance with the World Medical Association Helsinki Declaration and all applicable national laws and it was approved by the institutional review board, the United Ethical Review Committee for Research in Psychology (EPKEB).

### Stimuli

Two continuous Hungarian speech streams were presented simultaneously to the participants from two spatially separate loudspeakers. Each speech segment lasted of ca. 6 min duration (mean duration: 352.15 s, SD: 9.34; mean word number per segment: 636.41, SD: 84.87; mean number of phonemes per word: 6.48, SD: 0.29). Speech material was read by professional actors (two male speakers) recorded at 48 kHz with 32-bit resolution in the same room where the experiment was conducted and approximately from the same location where the loudspeakers were placed during the experiment. Thus room acoustics effects did not differ between recording and the experimental setup. Each loudspeaker corresponded to only one speaker. Thus participants received the same speech stream from the same location as it was recorded. One of the speakers (and correspondingly, one of the voices) was designated as the target of task (target stream), the other served as the distractor (distractor stream). The arrangement of the target and distractor streams was constant throughout the experiment: the target stream was always presented form the left loudspeaker with a fixed loudness level (∼70 dB SPL, measured at the participant’s head), whereas the loudness of the distractor speech stream (presented from the right loudspeaker) varied in five steps. The distractor-minus-target loudness difference was −10 dB (moderately softer distractor condition), −5 dB (slightly softer distractor condition), 0 dB (equal loudness condition), +5 dB (slightly louder distractor condition), +10 dB (moderately louder distractor condition). Consequently, five experimental conditions were created which were presented in 20 blocks (each condition received 4 blocks to avoid very long blocks). Speech material was news articles selected from Hungarian news websites. Articles were checked for correct-grammar, natural text flow, avoiding the use of garden-path sentences. They contained information not generally well-known. Altogether 40 articles were selected and presented in pairs, 20 of them where designated as target and the other 20 as distractor resulting in the 20 stimulus blocks. The 20 target and 20 distractor streams were the same for all participants.

Each speech stream contained numerals (45–57 each, *M* = 50.7, *SD* = 2.7) with 2–4 syllables, which served as target events within the attended stream. Only numerals indicating the quantity of something within the context of the text were valid targets. For example, in Hungarian, the indefinite article (“egy”) is the same as the word “one.” This word, when used as an article, did not count as a numeral. There are also words, such as the Hungarian word for moonflower or daisy (“százszorszép” – literally translated as “hundred-times-beautiful”), which have a numeral as a component. These were also not regarded as numerals. Numerals in the target stream are denoted as “target numerals,” while numerals in the distractor stream are denoted as “non-target numerals.” Syntactic violations were presented in the distractor stream only (“non-target syntactic violations”) and served as control events. Szalárdy et al. (2018) found that syntactic violations are not processed in the unattended stream. Therefore they can be used to indicate whether the non-target stream was attended or not (see Supplementary Material) for the description of syntactic violations.

### Procedure

Participants set in a sound-attenuated and electrically shielded room at the Research Centre for Natural Sciences, HAS, Budapest, Hungary. Two Mackie MR5 mk3 Powered Studio Monitor loudspeakers were placed at equal distance from them, positioned symmetrically at 30° left and right from the midline, with 200 cm distance from the participant’s head. Additionally, a 23″ monitor was placed at 195 cm in front of the participant, showing an unchanging a fixation cross (“+”) during the stimulus blocks. Participants were instructed to avoid eye blinks and other muscle movements and to watch the fixation cross while listening to the speech segments.

**FIGURE 1:**
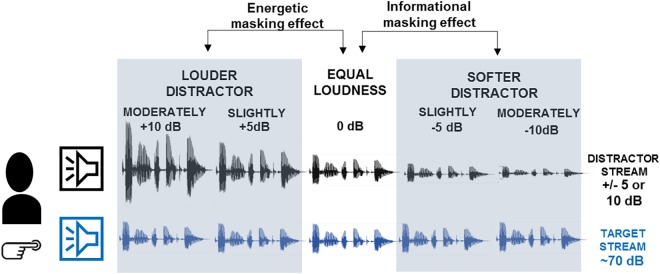
Schematic illustration of the experimental design. Participants listened to two concurrent speech streams presented from two spatially separate loudspeakers. The left stream (labeled by the blue loudspeaker) was designated as the target on which participants performed the numeral-detection and the content-tracking task. The right stream (labeled by black loudspeaker) served as distractor. The amplitude of the speech signals illustrates the loudness of the two speech streams in the different conditions. Based on the loudness difference between the target and distractor streams, five conditions were presented: moderately louder distractor, slightly louder distractor, equal loudness, slightly softer distractor, moderately softer distractor. The gray-shaded panels mark the expected impacts of the loudness difference: informational masking effects have been tested by comparing across the slightly and moderately softer distractor and the equal loudness conditions, whereas energetic masking effects across the slightly and moderately louder distractor and the equal loudness conditions.

Speech segments were presented by Matlab R2014a software (Mathworks Inc.) on an Intel Core i5 PC with ESI Julia 24-bit 192 kHz sound card connected to the loudspeakers. For each stimulus block, participants performed two tasks on the target speech segment (Figure 1). In the “numeral detection task,” participants were instructed to press a hand-held response key with their right thumb as soon as they detected the presence of a numeral word (target events, see above). In addition, a “content tracking task” was employed: listeners were informed that at the end of the stimulus block, they will be asked five questions regarding the contents of the target speech segment. The test consisted of five multiple-choice questions with four possible answers. Each question corresponded to one piece of information that appeared within the target speech segment. The experimenter read the question and the four possible answers and the listener was asked to verbally indicate the correct answer. The experimenter noted the participant’s choice and followed up with a request for confidence judgment with four alternatives: “I don’t remember I was just guessing,” “I am not sure, but the option I chose sounded familiar: I think I heard it during the last block,” “I am sure; I remember having heard it during the last block,” “I know the answer from some other source.” The confidence judgment was then recorded by the experimenter. The two concurrent tasks served complementary purposes in directing the listener’s attention: Whereas the tracking task required listeners to integrate information over longer periods of time and to fully process the target speech segments, the detection task ensured that attention was continuously focused on the target speech segment. Further, the detection task is compatible with similar tasks used in previous ERP studies (e.g., Ihlefeld and Shinn-Cunningham, 2008), allowing us to compare performance and ERP results with them. In contrast, the tracking task is more similar to what one does in everyday life situations. Performance in this task provides information about how listeners overall adapted to dynamically changing masking effects on a target speech stream.

The stimulus blocks were presented in pseudorandomized order: in the first half of the experimental session (blocks 1–10), each condition was presented two times in random order with the restriction that the same condition was not immediately repeated; in the second half of the session (blocks 11–20) conditions were presented in reversed order with respect to the first half. Participants were allowed to take a break during the experiment after each stimulus block any time they needed one, and there was a longer mandatory break after the 10th stimulus block. Altogether, the experiment lasted ca. 4 h.

### Data Analysis

#### Behavioral Data

Responses for the numeral-detection task were initially collected within the time window of 0–2000 ms from target word onsets. In order to eliminate the responses presumably not corresponding to targets (too early and too late responses), responses falling outside the 5–95% of all measured responses were rejected, resulting in an effective hit response window of 422–1646 ms. From the accepted responses, log-normalized hit reaction times (RT) were calculated for each participant and condition; note that the responses from the 4 blocks of the same condition were collapsed. *d*‘ values (the standard measure for detection sensitivity; Green and Swets, 1988) were calculated from the ‘hits’ and the ‘misses’ (the latter corresponding to the number of target events with no valid response), while ‘correct rejections’ and ‘false alarms’ were calculated by measuring hits and misses for non-target numerals (in the distractor stream) applying same time window as for hits.

Recognition performance in the content-tracking task was calculated as the percentage of correct responses pooled across stimulus blocks, separately for each participant and condition. The sensitivity of the measurement was increased by eliminating items (questions) above 95% or below 30% of the overall correct response rate (collapsed across participants and conditions). Correct responses where the confidence judgment was “I know the answer from some other source” were also eliminated from the analysis. Other confidence judgment data was not considered.

Statistical analysis was performed by analysis of variance (ANOVA) with the factors of LOUDNESS (moderately softer vs. slightly softer vs. equal vs. slightly louder vs. moderately louder distractor) separately for hit RT, *d*’, hit number, false alarm number, and recognition performance. All behavioral variables were tested whether they satisfy the assumptions of ANOVA by chi-square test, and none of them was significantly different from the normal distribution (*p*>0.05, all). The alpha level was set at 0.05. All significant results are reported. Greenhouse–Geisser correction of sphericity violations was employed where applicable and the ε correction factor is reported together with the η^2^ effect size. Statistical analysis was performed by STATISTICA 13.1 software.

#### EEG Data

##### Recording and preprocessing

Continuous EEG was recorded (1 kHz sampling rate and 100 Hz online low-pass filter) from a few seconds before the beginning to a few seconds after the end of the stimulus blocks using a BrainAmp DC 64-channel EEG system with actiCAP active electrodes (Brain Products GmbH). EEG signals were synchronized with the speech segments by matching an event trigger marked on the EEG record to the concurrent presentation of a beep sound in the audio stream (1 s before the speech segment commenced) with <1 ms accuracy. Electrodes were attached according to the extended International 10/20 system with an additional electrode placed on the tip of the nose. For identifying eye-movement artifacts, two electrodes were placed lateral to the outer canthi of the two eyes. Electrode impedances were kept below 15 kΩ. The FCz electrode served as an online reference.

Continuous EEG data was filtered with a 0.5–80.0 Hz Kaiser bandpass-filter and a 47.0–53.0 Hz Kaiser bandstop filter (the latter for removing electric noise; Kaiser β=5.65, filter length 18112 points) using the EEGlab 11.0.3.1.b toolbox (Delorme et al., 2007). EEG data processing was performed by Matlab R2014a (Mathworks Inc.). Electrodes with a continuous or large number of transient artifacts were interpolated using the spline interpolation algorithm implemented in EEGlab. The maximum number of interpolated channels was two per participant. The Infomax algorithm of Independent Component Analysis (ICA) implemented in EEGlab was employed for eye-movement artifact removal (Delorme et al., 2007). Maximum 6 ICA components (approximately 10% of all components) constituting blink artifacts and horizontal eye-movements were removed via visual inspection of the topographical distribution and frequency contents of the components. Data were then offline re-referenced to the average of the electrodes.

##### Functional connectivity analysis

Two thousand forty-eight long epochs were extracted from the continuous EEG records. Only event-free epochs were retained: epochs including a numeral, syntactic violation, or button press were excluded from the analysis in order to remove event/target-related brain activity (such as target detection and motor preparation) from the analysis. Artifact rejection with a threshold of 100 μV voltage change was applied to the whole epoch, separately for each electrode. With the criterion of at least 90 artifact-free epochs for each participant and condition, one participant’s data were rejected from further analysis.

Preprocessed data were entered to a minimum norm estimate model for source-reconstruction (sLORETA developed by Pascual-Marqui et al., 2002) using the Brainstorm toolbox (Tadel et al., 2011). The protocol was based on the studies of (Baillet et al., 2001; Pizzagalli, 2007; Song et al., 2015; Huang et al., 2016; Stropahl et al., 2018). The default electrode locations were entered into the forward boundary element head model provided by the openMEEG algorithm (Gramfort et al., 2011); the head model was based on default anatomy derived from the MNI/Colin27 brain (Collins et al., 1998). The time-varying source signals were modeled in all cortical voxels where the dipole had a component perpendicular to the cortical surface. Thirty six cortical regions (18 from each of the left and the right hemisphere) were selected as regions of interest from a standardized parcellation scheme introduced by Klein and Tourville (2012) and the mean neuronal activity (current density) was calculated for these regions by averaging dipole strengths across voxels (Table 1). Because we lack individual anatomical information, the spatial down-sampling to 36 ROIs by sLORETA (a distributed dipole model which, unlike, e.g., Beamformer does not assume that the source activity can be described in terms of uncorrelated dipoles), which provides maximal smoothness of the source activity across the brain tissue allows a sufficiently reliable description of brain activity without *a priori* information about the generators. Using accurate head models and a large number of electrodes, the localization accuracy of EEG could be comparable to MEG as it was shown by Stropahl et al. (2018). Our evaluation showed that the source localization errors for each cortical region are less than 20 mm [reported by Tóth et al. (2019)]. Thus, there are only a few neighboring regions (such as Heschl’s gyrus or pars orbitalis, pars-triangularis and the rostral and caudal anterior cingulate gyrus) for which the reconstructed source activity could be ambiguous. We, therefore, do not draw conclusions basing on FCs that would refer to these regions.

**Table 1:** EEG source regions (second column) are summarized according to their corresponding anatomical region (first column) together with their abbreviation (third column).

Anatomical region	EEG source region	Abbreviation
*Frontal*	Precentral gyrus	PrCG
	Inferior frontal gyrus	IFG
	Rostral middle frontal gyrus	MFGr
	Caudal middle frontal gyrus	MFGc
	Superior frontal lobe	SFG
	Orbitofrontal lobe	OFC
*Temporal*	Superior temporal lobe	STC
	Middle temporal gyrus	MTG
	Inferior temporal gyrus	ITG
	Fusiform gyrus	FFG
*Cingular*	Posterior cingulate lobe	PCC
	Anterior cingulate lobe	ACC
*Parietal*	Postcentral gyrus	PoCG
	Paracentral gyrus	PCG
	Supramarginal gyrus	SMG
	Inferior parietal gyrus	IPG
	Superior parietal	SPG
	Precuneus	Pcun

For each epoch phase lag index was measured and phase synchronization was calculated (PLI; see Stam et al., 2007) between each pair of EEG source region in five frequency bands (delta: 0.5–4 Hz; theta: 4–8 Hz, lower alpha: 8–10 Hz, upper alpha: 10–12 Hz, beta: 13–30 Hz, gamma 30–70 Hz). PLI is expressed by:

PLI=|<sign[Δφ(t_k)]>|

where Δφ(t_k) refers to the time series of phase differences (t) calculated over all *k* = 1…N time points of a trial, sign refers to the signum function, <> refers to the mean value and 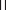 denotes the absolute value. PLI was calculated by BrainWave software (version 0.9.151.5)^1^ using the default setting with a gain set at 1. Random (minimum connectivity strength) and constant phase synchrony (maximum connectivity strength) is expressed by the PLI as 0 and 1, respectively. 36 × 36 FC matrices (consisting of PLI values) were calculated for each epoch which was then averaged for each participant separately for each frequency band, and condition. Visualization of FCs on circular graph plots was performed by a Matlab function developed by Paul Kassebaum^2^. Visualization of the results of the FC analysis over the cortical surface was performed by the BrainNet Viewer toolbox (Xia et al., 2013). For visualization of the cortical surface, the BrainMesh_ICBM152 surface template was applied to the nodes representing the cortical gyri as located by their standard MNI coordinates.

Statistical analysis was performed by the Network-Based Statistic (NBS) software package, developed for testing hypotheses about the human connectome (Zalesky et al., 2010). The NBS method exploits the tendency for experimental effects involving brain connectivity to exhibit specific topological characteristics that could not occur by chance in the absence of an effect. For the current data set, FC strength was tested separately for the six frequency bands. FC strength data were separately averaged across the slightly and moderately softer distractor conditions and across the slightly and moderately louder distractor conditions because NBS can best perform pairwise comparisons. Thus, the effects predominantly due to ENERGETIC MASKING were tested by comparing the FC networks between louder distractors and equal loudness, while those predominantly due to INFORMATIONAL MASKING were tested by comparing the FC networks between softer distractors and equal loudness.

Connections exceeding a chosen threshold (separately tested for each F threshold between 3 and 10) with the *F* test statistic have been first collected tested. The *F-values* were computed for each connection between the contrasted conditions. The number of all possible connections between the 36 regions was *N* = 630, given by the following equation: *N* = (36 × 35)/2. The NBS algorithm then identified distinct networks within the set of supra-threshold connections. Networks are defined as fully connected graphs (all nodes carrying at least one connection) that consists of only supra-threshold edges. Permutation-based mass univariate statistical testing was used on each identified network to set a family wise error corrected p-value for each network: 10000 random supra-threshold networks were created by repeatedly permuting the condition FC strength vectors, separately for each participant. The size (number of edges) of the largest network extracted from each permutation formed the distribution against which the original supra-threshold networks were tested (separately for each 3 ≥ F ≥ 10 threshold and statistical contrast). Networks with a size falling into the highest 5% of the distribution were regarded as significant. The significant networks obtained with the highest threshold were selected. The final threshold was set by determining the maximum value that still resulted in at least one significant network for the given contrast, separately for the six EEG frequency bands. Next, within each network, the edges were ordered according to the size of the connectivity strength difference between the contrasted conditions, and the 32 edges (proximately 10% all possible edges) with the highest difference were submitted to a *post hoc* pairwise *t-test*. Only edges with a significant (α=0.05) difference were selected for interpretation, as these characterize the largest FC strength difference for the contrast tested. Finally, depending on the direction of the difference between the two contrasted conditions, the edges were separated into two groups. Thus, when testing the effects of energetic masking, the edges that showed significantly higher FC strength for louder distractors relative to the equal loudness condition formed one group, whereas the ones showing the opposite effect, the other group; when testing informational masking the same distinction was employed for softer distractors and the equal loudness condition.

Finally, repeated measures contrasts were conducted as *post hoc* analyses comparing the FC networks emerging from the NBS analysis across the equal loudness, slightly and moderately louder distractor conditions and separately across the equal loudness, slightly and moderately softer distractor conditions in search for edges with monotonically increasing or decreasing FC strength along the three conditions.

Pearson correlation was calculated between the average connectivity strength difference of the networks emerging from the two LOUDNESS contrasts (energetic and information masking) and the difference in the different behavioral measures (*d*’, hit rate, false alarm rate, RT, and recognition performance) between the same two conditions (for more details, see Supplementary Material).

##### ERP data analysis

For analyzing the ERP responses, epochs were extracted from the continuous EEG record between −200 and +2200 ms relative to the onset of numerals and syntactic violations. Baseline correction was based on the −200–0 ms time window. Epochs exceeding the threshold of ±100 μV change throughout the whole epoch were rejected, separately for each electrode. For each event, the number of epochs retained after artifact rejection was at least 61 (*M*: 152.73, *SD*: 16.7 for numerals, *M*: 78.87, *SD*: 3.25 for syntactic violation) except for syntactic violations in the moderately softer distractor condition (*M*: 39.85, *SD*: 1.97).

As expected, target events elicited the N2b and P3b components with a parietal maximum for both. The time window for measuring the amplitude of these components was determined by the peak with the maximal amplitude and the duration of the component at the Pz electrode. The N2b amplitude was thus measured from the 120–280 ms post-stimulus time window, while the P3b amplitude between 550 and 750 ms. For numerals in the distractor stream, the time-windows were set the same as the corresponding ones in the target stream. The N400 component for non-target syntactic violations was measured at 350–550 ms relative to the stimulus onset, at the Pz electrode site (Szalárdy et al., 2018).

Amplitudes for target and non-target numerals were compared using repeated measure ANOVAs, with the factors of ATTENTION (target, distractor) × LOUDNESS (moderately softer vs. slightly softer vs. equal loudness vs. slightly louder vs. moderately louder distractor), separately for the N2b and P3b components. Statistical analysis of the N400 components was performed by one-tailed *t-test*. For the statistical analysis, STATISTICA 13.1 software was employed and post-hoc tests were conducted by Tukey’s HSD method. Again, Greenhouse-Geisser correction of sphericity violations was employed where applicable and the ε correction factor is reported together with the η^2^ effect size.

## Results

### Behavioral Results

**FIGURE 2:**
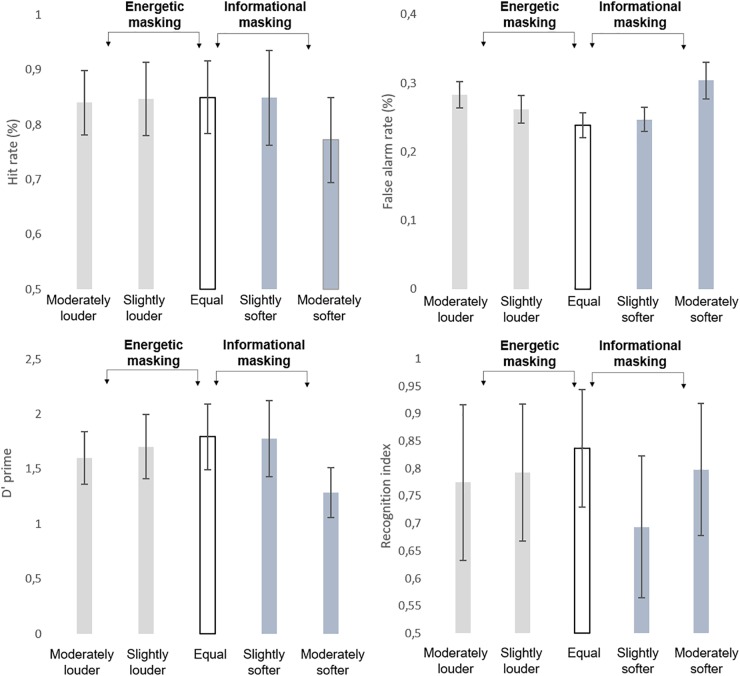
Group average (*N* = 27) performance in the detection task indexed by hit rate (HIT %, **top left panel**), false alarm rate (FALSE ALARM %; **top right panel**), detection sensitivity (*d*’, **bottom left panel**), and performance in the recognition task (recognition index, **bottom right panel**). Standard errors of the mean are shown for each data point.

A main effect of LOUDNESS (*F*_4,104_ = 10.595; ε = 0.900, *p*<0.001; $\eta_{p}^{2}$ = 0.289) was found on recognition performance (see averages and standard deviations on Figure 2). Tukey’s HSD *post hoc* comparison showed that in the slightly softer distractor condition recognition performance was significantly lower, than with any other loudness parameter (*p* < 0.01, each). None of the other conditions differed from each other.

Analysis of log-normalized (RT’s revealed no significant main effect (*p* > 0.9). The *d*’ values (see Figure 2) yielded a significant main effect of LOUDNESS (*F*_4,104_ = 72.683; ε = 0.844; *p* < 0.001; $\eta_{p}^{2}$ = 0.737). The post-hoc test revealed that participants’ performance was significantly lower in the moderately louder and moderately softer distractor conditions than in the rest of the conditions (*p* < 0.05, at least). These two conditions also differed from each other (*p* < 0.001).

Significant main effects of LOUDNESS were found on the hit (*F*_4,104_ = 28.802; ε = 0.821; *p*<0.001; $\eta_{p}^{2}$ = 0.526) and false alarm rates (*F*_4,104_ = 60.14; ε = 0.788; *p* < 0.001; $\eta_{p}^{2}$ = 0.698). *Post hoc* tests revealed that the hit rate was significantly lower in the moderately softer distractor condition than in all other conditions (*p* < 0.001, each), whereas a significant difference was found between all but the slightly softer distractor and equal loudness conditions (*p* < 0.05, at least), which showed the lowest false alarm rates. No significant correlation occurred between the behavioral variables and the average of the FC strength values (*p* > 0.05, all).

### EEG Functional Connectivity Networks

Statistical contrasts embedded within the network identification procedure revealed EEG functional networks affected by ENERGETIC MASKING (equal loudness vs. louder distractor) in the lower alpha and gamma and INFORMATIONAL MASKING (equal loudness vs. softer distractor) in the theta and beta frequency bands. For the delta, and high alpha bands, no significant network was found even using the lowest threshold (*F* = 3). Supplementary Table 3 shows the summary of the node degrees (the number of connections within the EEG networks showing a significant ENERGETIC MASKING or INFORMATIONAL MASKING effect), separately for each brain area.

#### The Effects of ENERGETIC MASKING on EEG Functional Connectivity

**FIGURE 3:**
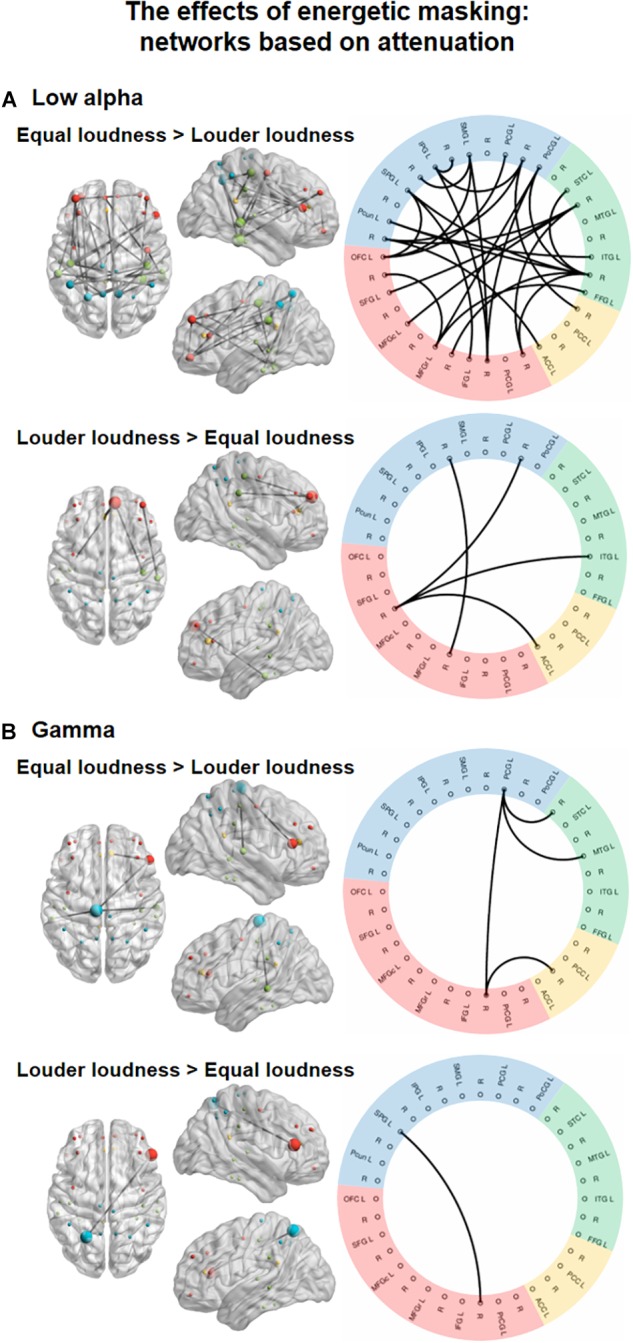
Functional networks in the low alpha (8–10 Hz, **A**) and gamma (30–70 Hz, **B**) EEG bands showing the significant effect of ENERGETIC MASKING (equal loudness vs. louder distractors). The left panels show the significant networks on a plot of the cortical surface (top, left, and right view). Colored dots (red – frontal, yellow – cingular, green – temporal, blue–parietal cortex) mark the spatial locations of the EEG sources reconstructed for cortical regions (nodes) in MNI space. The size of the node represents the degree (number of connections within the network) of each node (see Supplementary Table 3). The right panels show the circular graph representation of the significant network edges. Colors represent the lobe to which the nodes belong (same as described for the cortical surface plots). Brain regions are listed with their abbreviations in Table 1; L stands for left and R for right.

Significant main effects of ENERGETIC MASKING manipulation were found in the low alpha, and gamma bands (Figure 3), one network, each.

In the low alpha band, a network (Figure 3, top panel; *K* = 5.2, the threshold used in the *F* statistics; *p* = 0.0299) comprising 27 edges connecting 26 nodes was found. These links were stronger for the equal loudness condition relative to louder distractors (*p* < 0.05, all). Nodes with the highest number of connections were FFG (*N* = 7), and PoCG (*N* = 6, see Supplementary Table 3). This network featured connections separately within the frontal, temporal, and parietal regions as well as longer-range fronto-parietal, fronto-temporal and parieto-temporal links, and two links between the parietal and cingular areas. Another network consisting of 4 edges connecting 6 nodes, higher FC strength for louder distractors relative to the equal loudness condition (*p*<0.05, all) was also identified. This network was mainly included the connections of the left SFG with other temporal, parietal, and cingular areas, in the gamma frequency band, a network (Figure 3, bottom panel; *K* = 9.7; *p* = 0.0042) of 4 edges connecting 5 nodes had stronger FC values (*p* < 0.05, all) in the equal loudness condition than with louder distractors. These edges connected the parietal and temporal, frontal and cingular, and frontal and parietal areas, mediated mainly by the left PCG. Only one connection connecting frontal and parietal areas (*p* < 0.05) was significantly stronger with louder distractors compared to the equal loudness condition.

**FIGURE 4:**
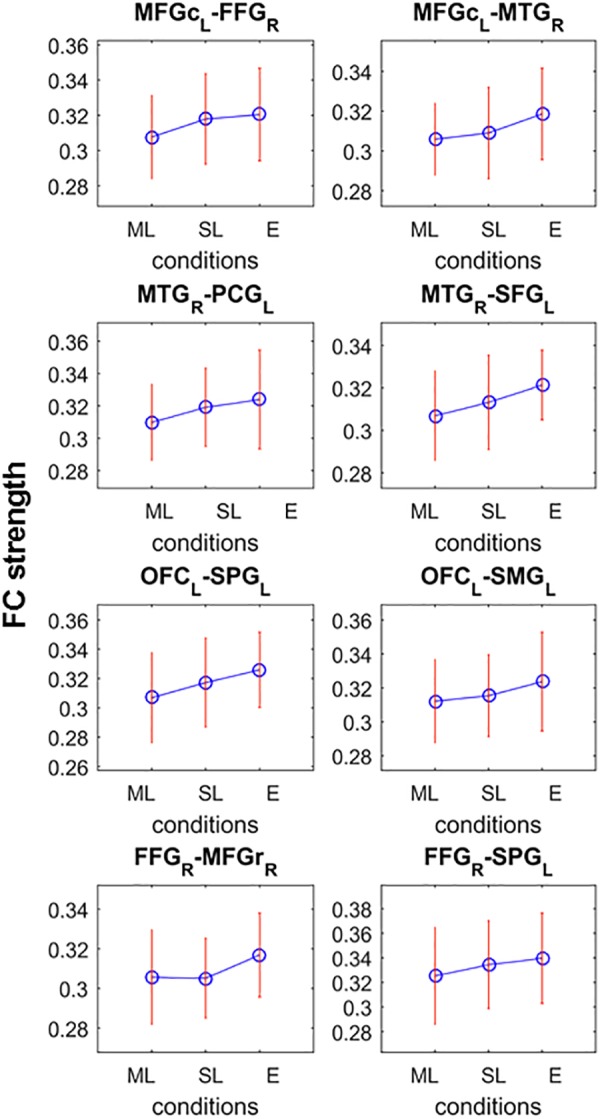
Significant network of the post-hoc analysis testing monotonic FC strength behavior as a function of loudness difference. Connections possess significant difference between at least two loudness level which the criteria that the FC strength (shown on *y-axes*) monotonically increase (ML, moderately louder distractor; SL, slightly louder distractor; E, equal loudness) for all but the last two nodes (FFG-MFGr and FFG-SPG).

*Post hoc* analyses searching for networks (*K* = 4.8; *p* = 0.0424) showing monotonic FC strength behavior as a function of loudness revealed a significant network in the low alpha frequency band comprising 8 edges in 6 of which the strength of the connectivity between the regions monotonically and significantly increased from the moderately louder distractor through the slightly louder distractor to the equal loudness condition (*p* < 0.05 for all 6 connections; see Figure 4). Two edges did not fulfill the criteria of significant monotonic behavior and were therefore excluded. The remaining network of 6 edges connecting 8 nodes features connections between the frontal and temporal, frontal and parietal, and temporal and parietal regions.

#### The Effects of INFORMATIONAL MASKING on EEG Functional Connectivity

**FIGURE 5:**
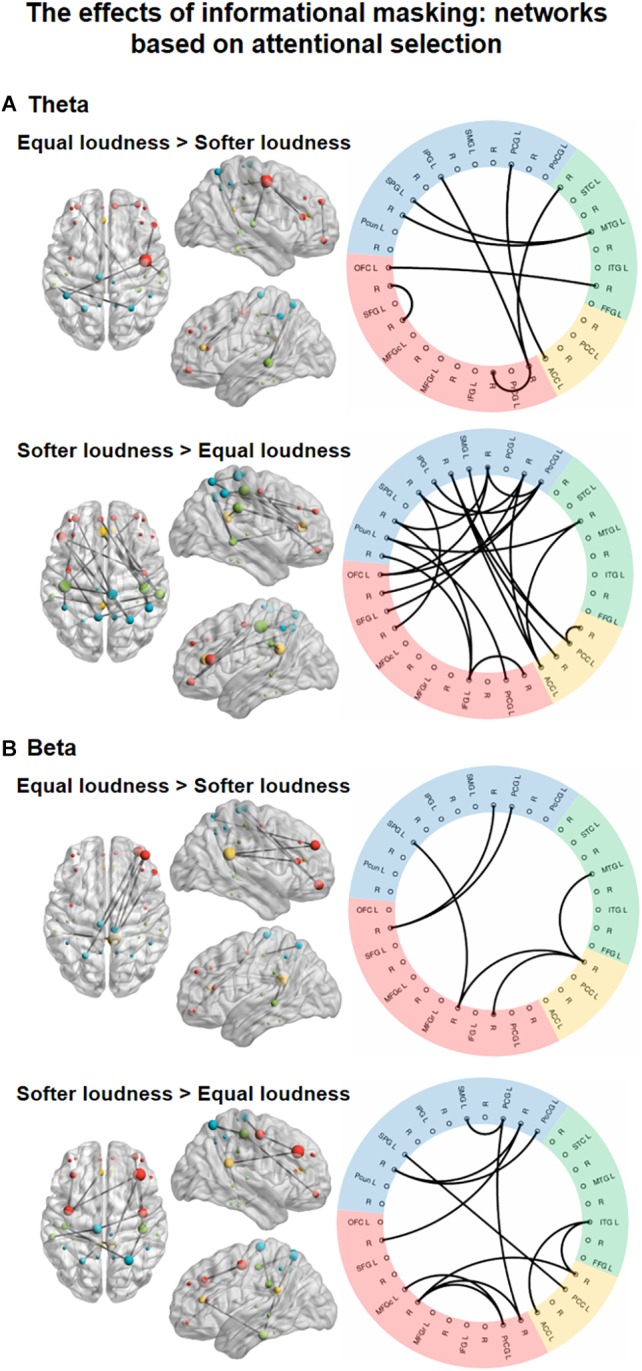
Functional networks in the theta (4–8 Hz, **A**) and beta (13–30 Hz, **B**) EEG bands showing the significant effect of INFORMATIONAL MASKING (equal loudness vs. softer distractors). The left panels show the significant networks on a plot of the cortical surface (top, left, and right view). Colored dots (red – frontal, yellow – cingular, green – temporal, blue–parietal cortex) mark the spatial locations of the EEG sources reconstructed for cortical regions (nodes) in MNI space. The size of the node represents the degree (number of connections within the network) of each node (see Supplementary Table 3). The right panels show the circular graph representation of the significant network edges. Colors represent the lobe to which the nodes belong (same as described for the cortical surface plots). Brain regions are listed with their abbreviations in Table 1; L stands for left and R for right.

Significant main effects of INFORMATIONAL MASKING manipulation were found in the theta-, and beta-band connectivity (Figure 5), one network, each.

In the theta band, a network (Figure 5, top panel; *K* = 5.1; *p* = 0.0372) comprising 8 edges connecting 13 nodes was found with stronger FC (*p* < 0.05, all) during the equal loudness condition relative to softer distractors. This network featured connections between frontal and temporal, frontal and parietal, and temporal and parietal regions with an additional link between the parietal and cingular areas and links within the frontal areas. A larger network of 21 edges connecting 22 nodes (*p* < 0.05, all) had stronger FC with softer distractors relative to the equal loudness condition. These connections were observed separately within the frontal, cingular, and parietal areas as well as for longer-range fronto-parietal, parieto-temporal as well as parietal-cingular, temporal–cingular links. The node with the highest number of connections was the PoCG (*N* = 7, see Supplementary Table 3).

In the beta frequency band, a network (Figure 5, bottom panel; *K* = 5.8; *p* = 0.0475) of 6 edges connecting 8 nodes had stronger FC (*p* < 0.05, all) in the equal loudness condition than with softer distractors. This network was composed of links between the frontal and parietal, frontal and temporal, and cingular and temporal areas. A network of 12 edges connecting 15 nodes showed stronger FC with softer distractors compared to the equal loudness condition (*p* < 0.05). This network included links mainly within the frontal and parietal areas with a few links between frontal and cingular, temporal and cingular, and frontal and parietal areas.

Contrasting the moderately softer distractor, slightly softer distractor, and equal loudness conditions, the *post hoc* analyses revealed a significant network in the theta band comprising 30 edges. However, the direction of the connectivity strength did not show monotonically increasing or decreasing trend except for four edges (ACC-PCG, PCG-PCG, PrCG-Pcun, IFG-SPG) see Supplementary Figure 1.

### ERP Results

**FIGURE 6:**
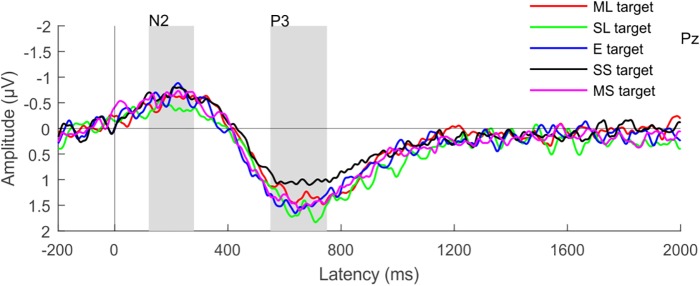
Group-average (*N* = 27) parietal (Pz) ERP responses elicited by target numerals. Color represents the different loudness conditions (ML, moderately louder; SL, slightly louder; E, equal loudness; MS, moderately softer; SS, slightly softer distractor); the N2b and P3b latency ranges are shown by gray rectangles.

Target numerals elicited the N2b and P3b components (Figure 6). The amplitude of both components was highest at the Pz electrode with centro-parietal scalp distributions. The ANOVA tests yielded significant main effects of ATTENTION for both the N2b and P3b: target numerals elicited significantly larger components than non-target numerals (for N2b: *F*_1,26_ = 42.245; *p* < 0.001; $\eta_{p}^{2}$ = 0.619 and for P3b: *F*_1,26_ = 106.86; *p*<0.001; $\eta_{p}^{2}$ = 0.804). The ANOVAs of the P3b amplitudes for target and non-target numerals revealed also significant main effect of LOUDNESS (*F*_4,104_ = 5.446; ε = 0.903; *p* < 0.001; $\eta_{p}^{2}$ = 0.173). Although no significant interaction was found between the LOUDNESS and ATTENTION factors for the P3b, there was a strong tendency (*p* = 0.057). As non-target numerals did not elicit the P3b response (supported by one-tailed *t-test*, *p* > 0.073, at least) separate ANOVAs were conducted for the P3b amplitude for target numerals with the factor of LOUDNESS, which revealed significant main effect (*F*_4,104_ = 5.446; ε = 0.8558; *p* < 0.001; $\eta_{p}^{2}$ = 0.1667). *Post hoc* comparisons showed that the target numerals in the slightly softer distractor condition elicited significantly lower P3b amplitudes than in the rest of the conditions except for the moderately louder distractor condition. None of the other conditions differed from each other. We did not find signs of syntactic reorganization in the distractor speech streams indicated by the lack of N400 for the syntactic violations. Nor did we find signs of numeral detection in the distractor stream as indicated by the lack of significant N2 (*p* > 0.075, at least; one-tailed *t-tests*; see Supplementary Figure 2).

## Discussion

In the current study, we measured task performance, ERP responses, and EEG-based FC for assessing the effects of energetic and informational masking in a dichotic listening task. Participants were presented with two concurrent speech streams, one of them assigned as the target for the listener’s task while the other as a distractor. Because both streams comprised continuous natural speech, the distractor always exerted informational masking on the target, whereas energetic masking was realized by increasing the intensity level of the distractor compared to the target. The lack of significant ERP responses to distractor numerals and syntactic violations confirmed that the distractor streams were indeed unattended (see Supplementary Figure 2). Although the intensity of the distractor stream was increased gradually in equal steps, the behavioral, ERP, and FC results showed clear non-monotonic effects, thus distinguishing between informational and energetic masking. Behavioral results showed that the recognition index, hit rate, and false alarm rate were affected differently by the two types of masking. In accordance with our hypothesis regarding the local vs. global nature of the two types of masking effects, target detection (characterized by *d*’ and the false alarm rate) was affected both when energetic and when information masking was assumed to be dominant, whereas content tracking (characterized by the recognition index) was mainly affected when information masking was expected to be dominant. In contrast to the study of Ihlefeld and Shinn-Cunningham (2008), we found the best behavioral performance was found in the equal loudness condition (regarding all behavioral indicator). Comparing the louder and softer distractor conditions with the equal loudness condition different patterns of performance indices were found for informational and energetic masking. At the same time, the FC data showed different activity patterns of neural networks for the two types of masking. While energetic masking was assumed to be dominant, we found significant networks in the low alpha and gamma frequency band. In contrast, when informational masking was dominant, theta- and beta-band networks were observed. Together with the FC changes, ERPs for target events showed different patterns for informational and energetic masking.

Somewhat surprisingly, RT was not sensitive to the applied task manipulations. This is not the first time a similar result has been obtained. Benett et al. (2012) employed three maskers: speech-shaped interrupted noise, speech-shaped continuous noise and four-talker babble noise. They found that with the four-talker babble noise the RT was longer than in the continuous and interrupted noise conditions, while the latter two did not differ from each other. In Benett and colleagues’ experiment, the speech content was identical in all conditions and energetic masking did not affect the RT responses at least in the intensity range tested. Rather, only the hit rate and the false alarm rate were affected, similarly to the study of Ihlefeld and Shinn-Cunningham (2008). Thus RT does not appear to be sensitive to the type of the masking effect.

In the following, the different effects are separately discussed.

### The Effects of Energetic Masking

We found that *d*’ was significantly lower in the moderately louder distractor condition than in the slightly louder and equal loudness conditions, whereas recognition performance was not affected. This suggests that energetic masking impaired short-term segmentation of the target speech stream to some degree (although not so much that the two streams could not be segregated at all). A separate analysis of the hit and false alarm rates revealed that the decreasing detection sensitivity was due to increasing false alarm rates, whereas hit rates were not affected by increasing the loudness of the distractor. Thus, with increasing energetic masking (increasing distractor loudness), participants were more likely to respond to numerals in the distractor stream, whereas detection of the target numerals was not significantly affected. Brain FC analysis revealed that the performance effects were accompanied by weaker low-alpha band synchronization compared to the equal loudness condition, whereas the ERPs did not show significant changes while energetic masking was increased.

The low-alpha band network possessed a large number of connections, which were stronger in the equal loudness compared to the louder masker condition. This network included nodes in the frontal (OFC, SFG, MFGc, MFGr, IFG, PrCG), parietal (PoCG, PCG, SMG, IPG, SPG, Pcun), temporal (FFG, ITG, MTG, STG), and cingular (ACC, PCC) brain regions. This type of global alpha synchronization is usually associated with sustained attention (Makeig and Jung, 1995; Braboszcz and Delorme, 2011) and attentional suppression of the distractor stimuli (Foxe and Snyder, 2011). This result together with the significantly increased false alarm rate suggests that attentional selection was less successful as energetic masking increased, whereas linking speech elements across time was probably preserved sincethe hit rate and recognition performance was not affected. Our results show that inhibition was more effective in the equal loudness condition, which matches the performance data. The *post hoc* test revealed that the strength of the connectivity monotonically increased form the moderately louder distractor toward the equal loudness condition. This indicates that these conditions were quantitatively different from each other with monotonically changing connectivity strength as energetic masking increased.

Together with the suppression of the irrelevant information mediated by networks operating in the low alpha band, gamma phase synchronization was also stronger in the equal loudness compared to the louder distractor conditions. The nodes were more strongly coupled with each other within temporal areas, involving the medial temporal gyrus (MTG) and superior temporal cortex (STC) and between temporal areas and the postcentral gyrus (PCG) and inferior frontal gyrus (IFG), also involving the anterior cingulate cortex (ACC). The ACC is presumed to serve as a relay station between frontal speech producing regions found in the IFG and it plays a critical role in the control of attention, executive processes, word generation, and memory (Hopfinger et al., 2000; Woldorff et al., 2004). Gamma enhancement has been associated with enhanced attention to the sensory events (Ahveninen et al., 2013; Potes et al., 2014). Therefore, our results suggest that the observed gamma band network may be involved in stream selection and target detection.

In summary, energetic masking elicited stronger local FC and large scale fronto-parietal connectivity in the low alpha band together with the increased coupling of sensory and perceptual areas (such as temporal and parietal cortices) through gamma-band oscillations. The present FC results together with the behavioral performance are compatible with our hypothesis that solving the selective listening task while energetic masking is dominant involved selectively enhancing the sensory input mediated by gamma-band oscillatory networks while suppressing the irrelevant information by networks operating in the low alpha band.

### The Effects of Informational Masking

Our results showed that *d*’ was significantly lower in the moderately softer distractor than in the slightly softer and equal loudness conditions. Hit rate was lowest in the moderately softer distractor condition, whereas the other conditions did not significantly differ from each other. False alarm rate was highest in the moderately softer loudness condition, significantly higher than in the equal loudness and slightly softer distractor conditions. Thus in contrast to the conditions where energetic masking dominated, informational masking affected both the hit and the false alarm rate. Further, the tracking-task (recognition) performance was lowest with the slightly softer distractor stream, a possible effect of information masking.

Ihlefeld and Shinn-Cunningham (2008) found that false alarms (they called them masker errors) were more likely when the loudness of the target and the distractor was equal compared to when modest loudness difference was applied between the streams. However, this only occurred when the two speech streams were co-located. Applying 90° HRTF difference between the two speech streams this effect disappeared and performance increased monotonically with increasing target loudness. In the present study, the two loudspeakers were placed at ±45° from the midline of the listener’s head. Thus, the current design was similar to Ihlefeld and Shinn-Cunningham’s 90° HRTF condition. Therefore, it is surprising that hit and false alarm rates were the worst in the moderately softer distractor condition. These results suggest that attentional selection was less successful in that condition compared to the equal loudness condition. It is also possible that participants failed to form links across the individual segments of the streams in the moderately softer condition. The FC and ERP results (see below) are more compatible with less successful attentional selection in the moderately softer distractor than in the equal loudness condition. Selective attention improves performance by enhancing gain on cortical activity related to the target, and/or suppressing the activity elicited by the distracting events (Mesgarani and Chang, 2012; Power et al., 2012; Golumbic et al., 2013). One possibility is that the gain of the suppression and facilitation changes between situations with dominant energetic vs. informational masking. Alternatively, is also possible that participants found the task easiest in the moderately softer condition (because it provided the best S/N ration between the attended and the distractor speech stream within the experimental session). As a consequence, they did not devote sufficient cognitive effort for filtering out the irrelevant information - in essence, they used these stimulus blocks to rest between the more challenging ones. Finally, it is also possible that listeners adapted to the louder distractor stream while remaining more sensitive to the softer ones. Watkins and Barbour (2011) found that non-monotonic neurons in auditory cortex tend to adapt to loud sounds while remaining sensitive for softer sounds. This and the previous explanation assume contextual effects in which the random-order mixing of the different distractor loudness conditions interacted with each other.

Recognition performance appeared to be independent of detection performance, as it was lowest in the slightly softer distractor condition in which the hit rate and false alarm rates were high. The pattern of P3b amplitudes was similar to that of the recognition index: significantly smaller P3b was found in the slightly softer loudness condition. Previous studies suggested that P3b is a correlate of resource allocation (Isreal et al., 1980), because of its sensitivity to target-probability (Kutas et al., 1977), stimulus intensity (Covington and Polich, 1996), the quality and task-relevance of a stimulus, and attention (Polich, 2007); in short: when more cognitive effort is needed for detecting targets. In addition, a recent visual detection study found enhanced P3b amplitude for targets in the presence of continuous distraction (Demeter et al., 2016). Thus, it possible that the significantly smaller P3b amplitude observed in the slightly softer distractor condition was due to the poorer allocation of attention. (Note, however, that this does not explain the difference between the slightly and moderately softer distractor conditions).

Informational masking increased synchrony in networks with different topographies in the theta and beta frequency bands. In the softer distractor condition, a primarily fronto-parietal network emerged in the theta band. This network involved several local connections within parietal (PoCG, PCG, SMG, IPG, SPG, Pcun) and frontal regions (OFC, SFG, IFG, PrCG), and longer-range connections between the frontal, parietal, and cingular (ACC, PCC) cortices. Only one connection was found with a temporal region (STC). In contrast, sparser connections were found stronger in the equal loudness than in the softer distractor condition. This network showed stronger temporal cortical involvement (FFG, MTG). Theta synchronization in the fronto-parietal attention network has been observed in several selective attention studies (Womelsdorf and Fries, 2007; Sauseng et al., 2008; Clayton et al., 2015) and it has been associated with both enhanced task performance and attentional fatigue. In relation to the current performance results, it is possible that the facilitation of sensory information processing for the attended stream was less effective in the informational masking conditions, because although connections were denser in the softer distractor conditions, the auditory sensory brain areas were almost absent from the networks showing stronger phase synchronization with softer distractors. In contrast, in the equal loudness condition, selective enhancement of auditory sensory information processing appeared to be mediated by fronto-temporal and temporo-parietal connections oscillating in the theta band. This result may explain the higher hit-rate in the equal loudness condition compared to the softer distractor conditions. The *post hoc* analysis revealed that there is no obvious monotonically increasing or decreasing tendency from the moderately softer distractor toward the equal loudness condition as was found for energetic masking in the low-alpha band. Thus, it is more feasible to suggest that the softer distractor conditions were qualitatively different from each other rather than quantitatively, which is compatible with the observed non-monotony of the different performance measures.

In the beta band, local connections have been found, particularly within parietal (PoCG, PCG, SMG, SPG) and frontal (OFC, MFGc, PrCG) regions and connections with the temporal (ITG) and cingular (ACC, PCC) regions, which were stronger for softer than equal-loudness distractors. In contrast, the equal loudness conditions possessed fewer, but longer-range connections between frontal (OFC, MFGr, IFG) and parietal (PCG, SPG), frontal and cingular (PCC), and frontal and temporal (MTG) brain regions. Although we had no hypothesis for the role of beta-band networks in informational masking, in accordance with previous studies, this beta-band synchronization can be interpreted as facilitating the processing of task-relevant stimuli and contributing to the prefrontal monitoring function (Miller and Cohen, 2001; Buschman and Miller, 2007). Although there were no clear regional differences between the networks which were stronger in the softer distractor than in the equal loudness condition, the presence of more local connections may indicate the involvement of the frontal and parietal control regions in informational masking. However, no relationship with detection task performance has been observed.

In summary, the present results suggest that in contrast to listening during strong energetic masking, the problem of performing the tasks during stronger informational masking may allocate mostly selective attention-dependent processes distributed in a widespread attention network. Specifically in line with our hypothesis informational masking elicited stronger theta band large-scale FC within the attentional control network centered around the hub region of the frontal cortex. In addition, we found a beta-band network sensitive to information masking, which may reflect the requirement of stronger prefrontal monitoring in this situation.

## Conclusion

The current study investigated speech processing (target detection and content analysis) in a selective listening task in the presence of energetic and informational masking. The behavioral, ERP and FC data suggests that the different masking effects induced different strategies and neural mechanisms for solving the task. We found that in the conditions in which energetic masking was dominant that the FC and behavioral results indicated suppression of the irrelevant information and facilitation of relevant sensory information. Suppressing distractors was probably mediated by a network operating in the low alpha band, whereas facilitation of target processing was accompanied by a gamma band network. In contrast, when informational masking had the higher hand, theta band synchronization became stronger, suggesting the activation of a wide-spread general attentional network. The most surprising result was that with softer maskers, target detection was not improved, rather, both target detection and distractor suppression became less effective. Various *post hoc* interpretations have been offered. However, they need to be distinguished and tested in future studies.

## Ethics Statement

The experiment was conducted in full accordance with the World Medical Association Helsinki Declaration and all applicable national laws and it was approved by the institutional review board, the United Ethical Review Committee for Research in Psychology (EPKEB).

## Author Contributions

OS analyzed the data including the behavioral and EEG analysis and statistical analysis and wrote up the manuscript. BT guided the functional connectivity analysis and contributed to the writing procedure. EG contributed to the ERP analysis. DF contributed to the behavioral analysis and functional network analysis. IW was leading the whole project, designed the study, and guided the analysis and writing procedure.

## Conflict of Interest Statement

The authors declare that the research was conducted in the absence of any commercial or financial relationships that could be construed as a potential conflict of interest.

## Footnotes

1 http://home.kpn.nl/stam7883/brainwave.html

2 http://www.mathworks.com/matlabcentral/fileexchange/48576-circulargraph

## References

[B1] Ahveninen J. Huang S. Belliveau J. W. Chang W. T. Hamalainen M. (2013). Dynamic oscillatory processes governing cued orienting and allocation of auditory attention. *J. Cogn. Neurosci.* 25 1926–1943. 10.1162/jocn_a_00452 23915050 PMC3788846

[B2] Akimoto Y. Kanno A. Kambara T. Nozawa T. Sugiura M. Okumura E. (2013). Spatiotemporal dynamics of high-gamma activities during a 3-stimulus visual oddball task. *PLoS One* 8:e59969. 10.1371/journal.pone.0059969 23555852 PMC3605370

[B3] Arbogast T. L. Mason C. R. Kidd G. JR. (2002). The effect of spatial separation on informational and energetic masking of speech. *J. Acoust. Soc. Am.* 112 2086–2098. 10.1121/1.1510141 12430820

[B4] Baillet S. Riera J. J. Marin G. Mangin J. F. Aubert J. Garnero L. (2001). Evaluation of inverse methods and head models for EEG source localization using a human skull phantom. *Phys. Med. Biol.* 46 77–96. 10.1088/0031-9155/46/1/306 11197680

[B5] Benett K. O. Billings C. J. Molis M. R. Leek M. R. (2012). Neural encoding and perception of speech signals in informational masking. *Ear Hear.* 33 231–238. 10.1097/AUD.0b013e31823173fd 22367094 PMC3292743

[B6] Braboszcz C. Delorme A. (2011). Lost in thoughts: neural markers of low alertness during mind wandering. *Neuroimage* 54 3040–3047. 10.1016/j.neuroimage.2010.10.008 20946963

[B7] Brancucci A. Penna S. D. Babiloni C. Vecchio F. Capotosto P. Rossi D. (2008). Neuromagnetic functional coupling during dichotic listening of speech sounds. *Hum. Brain Mapp.* 29 253–264. 10.1002/hbm.20385 17370343 PMC6871073

[B8] Brazdil M. Janecek J. Klimes P. Marecek R. Roman R. Jurak P. (2013). On the time course of synchronization patterns of neuronal discharges in the human brain during cognitive tasks. *PLoS One* 8:e63293. 10.1371/journal.pone.0063293 23696809 PMC3655978

[B9] Bregman A. S. (1990). *Auditory Scene Analysis: The Perceptual Organization of Sound.* Cambridge, MA: MIT Press.

[B10] Brungart D. S. Simpson B. D. Ericson M. A. Scott K. R. (2001). Informational and energetic masking effects in the perception of multiple simultaneous talkers. *J. Acoust. Soc. Am.* 110 2527–2538. 10.1121/1.1345696 11757942

[B11] Buschman T. J. Miller E. K. (2007). Top-down versus bottom-up control of attention in the prefrontal and posterior parietal cortices. *Science* 315 1860–1862. 10.1126/science.1138071 17395832

[B12] Carlyon R. P. (2004). How the brain separates sounds. *Trends Cogn. Sci.* 8 465–471. 10.1016/j.tics.2004.08.008 15450511

[B13] Clayton M. S. Yeung N. Cohen R. Kadosh R. (2015). The roles of cortical oscillations in sustained attention. *Trends Cogn. Sci.* 19 188–195. 10.1016/j.tics.2015.02.004 25765608

[B14] Collins D. L. Zijdenbos A. P. Kollokian V. Sled J. G. Kabani N. J. Holmes C. J. (1998). Design and construction of a realistic digital brain phantom. *IEEE Trans. Med. Imaging* 17 463–468. 10.1109/42.712135 9735909

[B15] Cooper N. R. Croft R. J. Dominey S. J. J. Burgess A. P. Gruzelier J. H. (2003). Paradox lost? Exploring the role of alpha oscillations during externally vs. internally directed attention and the implications for idling and inhibition hypotheses. *Int. J. Psychophysiol.* 47 65–74. 10.1016/S0167-8760(02)00107-1 12543447

[B16] Covington J. W. Polich J. (1996). P300, stimulus intensity, and modality. *Electroencephalogr. Clin. Neurophysiol.* 100 579–584. 10.1016/S0168-5597(96)96013-X 8980423

[B17] Daitch A. L. Sharma M. Roland J. L. Astafiev S. V. Bundy D. T. Gaona C. M. (2013). Frequency-specific mechanism links human brain networks for spatial attention. *Proc. Natl. Acad. Sci. U.S.A.* 110 19585–19590. 10.1073/pnas.1307947110 24218604 PMC3845177

[B18] Darwin C. J. (2008). Listening to speech in the presence of other sounds. *Philos. Trans. Soc. R. Lond. B. Biol. Sci.* 363 1011–1021. 10.1098/rstb.2007.2156 17827106 PMC2606793

[B19] Delorme A. Sejnowski T. Makeig S. (2007). Enhanced detection of artifacts in EEG data using higher-order statistics and independent component analysis. *Neuroimage* 34 1443–1449. 10.1016/j.neuroimage.2006.11.004 17188898 PMC2895624

[B20] Demeter E. De Alburquerque D. Woldorff M. G. (2016). The effects of ongoing distraction on the neural processes underlying signal detection. *Neuropsychologia* 89 335–343. 10.1016/j.neuropsychologia.2016.06.038 27378439 PMC5089870

[B21] Desimone R. Duncan J. (1995). Neural mechanisms of selective visual attention. *Annu. Rev. Neurosci.* 18 193–222. 10.1146/annurev.ne.18.030195.001205 7605061

[B22] Dombrowe I. Hilgetag C. C. (2014). Occipitoparietal alpha-band responses to the graded allocation of top-down spatial attention. *J. Neurophysiol.* 112 1307–1316. 10.1152/jn.00654.2013 24966295

[B23] Donchin E. Coles M. G. H. (1988). Is the P300 component a manifestation of context updating? *Behav. Brain Sci.* 11 357–374. 10.1017/S0140525X00058027 22974337

[B24] Fell J. Axmacher N. (2011). The role of phase synchronization in memory processes. *Nat. Rev. Neurosci.* 12 105–118. 10.1038/nrn2979<pmid< 21248789

[B25] Foxe J. J. Snyder A. C. (2011). The role of alpha-band brain oscillations as a sensory suppression mechanism during selective attention. *Front. Psychol.* 2:154. 10.3389/fpsyg.2011.00154 21779269 PMC3132683

[B26] Freyman R. L. Balakrishnan U. Helfer K. S. (2004). Effect of number of masking talkers and auditory priming on informational masking in speech recognition. *J. Acoust. Soc. Am.* 115 2246–2256. 10.1121/1.689343 15139635

[B27] Freyman R. L. Helfer K. S. McCall D. D. Clifton R. K. (1999). The role of perceived spatial separation in the unmasking of speech. *J. Acoust. Soc. Am.* 106 3578–3588. 10.1121/1.428211 10615698

[B28] Golumbic E. M. Z. Ding N. Bickel S. Lakatos P. Schevon C. A. McKhann G. M. (2013). Mechanisms underlying selective neuronal tracking of attended speech at a “cocktail party”. *Neuron* 77 980–991. 10.1016/j.neuron.2012.12.037 23473326 PMC3891478

[B29] Gramfort A. Papadopoulo T. Olivi E. Clerc M. (2011). Forward field computation with OpenMEEG. *Comput. Intell. Neurosci.* 2011:923703. 10.1155/2011/923703 21437231 PMC3061324

[B30] Green D. M. Swets J. A. (1988). *Signal Detection Theory and Psychophysics.* Los Altos, PA: Wiley

[B31] Hansen J. C. Dickstein P. W. Berka C. Hillyard S. A. (1983). Event-related potentials during selective attention to speech sounds. *Biol. Psychol.* 16 211–224. 10.1016/0301-0511(83)90025-X 6615954

[B32] Herrmann B. Henry M. J. Haegens S. Obleser J. (2016). Temporal expectations and neural amplitude fluctuations in auditory cortex interactively influence perception. *Neuroimage* 124 487–497. 10.1016/j.neuroimage.2015.09.019 26386347

[B33] Hopfinger J. B. Buonocore M. H. Mangun G. R. (2000). The neural mechanisms of top-down attentional control. *Nat. Neurosci.* 3 284–291. 10.1038/72999 10700262

[B34] Huang Y. Parra L. C. Haufe S. (2016). The New York Head-A precise standardized volume conductor model for EEG source localization and tES targeting. *Neuroimage* 140 150–162. 10.1016/j.neuroimage.2015.12.019 26706450 PMC5778879

[B35] Ihlefeld A. Shinn-Cunningham B. (2008). Spatial release from energetic and informational masking in a selective speech identification task. *J. Acoust. Soc. Am.* 123 4369–4379. 10.1121/1.2904826 18537388 PMC9014252

[B36] Isreal J. B. Chesney G. L. Wickens C. D. Donchin E. (1980). P300 and tracking difficulty: evidence for multiple resources in dual-task performance. *Psychophysiology* 17 259–273. 10.1111/j.1469-8986.1980.tb00146.x 7384376

[B37] Kerlin J. R. Shahin A. J. Miller L. M. (2010). Attentional gain control of ongoing cortical speech representations in a “Cocktail Party”. *J. Neurosci.* 30 620–628. 10.1523/Jneurosci.3631-09.2010 20071526 PMC2832933

[B38] Kidd G. Jr. Arbogast T. L. Mason C. R. Gallun F. J. (2005). The advantage of knowing where to listen. *J. Acoust. Soc. Am.* 118 3804–3815. 10.1121/1.2109187 16419825

[B39] Klein A. Tourville J. (2012). 101 labeled brain images and a consistent human cortical labeling protocol. *Front. Neurosci.* 6:171. 10.3389/fnins.2012.00171 23227001 PMC3514540

[B40] Klimesch W. Sauseng P. Hanslmayr S. Gruber W. Freunberger R. (2007). Event-related phase reorganization may explain evoked neural dynamics. *Neurosci. Biobehav. Rev.* 31 1003–1016. 10.1016/j.neubiorev.2007.03.005 17532471

[B41] Kutas M. McCarthy G. Donchin E. (1977). Augmenting mental chronometry: the P300 as a measure of stimulus evaluation time. *Science* 197 792–795. 10.1126/science.887923 887923

[B42] Liu Y. Bengson J. Huang H. Mangun G. R. Ding M. (2016). Top-down modulation of neural activity in anticipatory visual attention: control mechanisms revealed by simultaneous EEG-fMRI. *Cereb. Cortex* 26 517–529. 10.1093/cercor/bhu204 25205663 PMC4712792

[B43] MacDonald A. W. Cohen J. D. Stenger V. A. Carter C. S. (2000). Dissociating the role of the dorsolateral prefrontal and anterior cingulate cortex in cognitive control. *Science* 288 1835–1838. 10.1126/science.288.5472.1835 10846167

[B44] Makeig S. Jung T. P. (1995). Changes in alertness are a principal component of variance in the EEG spectrum. *Neuroreport* 7 213–216. 10.1097/00001756-199512000-00051 8742454

[B45] Mesgarani N. Chang E. F. (2012). Selective cortical representation of attended speaker in multi-talker speech perception. *Nature* 485 233–236. 10.1038/nature11020 22522927 PMC3870007

[B46] Miller E. K. Cohen J. D. (2001). An integrative theory of prefrontal cortex function. *Annu. Rev. Neurosci.* 24 167–202. 10.1146/annurev.neuro.24.1.167 11283309

[B47] Näätänen R. Simpson M. Loveless N. E. (1982). Stimulus deviance and evoked potentials. *Biol. Psychol.* 14 53–98. 10.1016/0301-0511(82)90017-5 7104425

[B48] Najafi M. McMenamin B. W. Simon J. Z. Pessoa L. (2016). Overlapping communities reveal rich structure in large-scale brain networks during rest and task conditions. *Neuroimage* 135 92–106. 10.1016/j.neuroimage.2016.04.054 27129758 PMC4915991

[B49] Pascual-Marqui R. D. Esslen M. K. Kochi K. Lehmann D. (2002). Functional imaging with low-resolution brain electromagnetic tomography (LORETA): a review. *Methods Find. Exp. Clin. Pharmacol.* 24(Suppl. C), 91–95. 12575492

[B50] Pizzagalli D. A. (2007). Electroencephalography and high-density electrophysiological source localization. *Handb. Psychophysiol.* 3 56–84. 10.1017/cbo9780511546396.003

[B51] Polich J. (2007). Updating p300: an integrative theory of P3a and P3b. *Clin. Neurophysiol.* 118 2128–2148. 10.1016/j.clinph.2007.04.019 17573239 PMC2715154

[B52] Polich J. Herbst K. L. (2000). P300 as a clinical assay: rationale, evaluation, and findings. *Int. J. Psychophysiol.* 38 3–19. 10.1016/S0167-8760(00)00127-6 11027791

[B53] Potes C. Brunner P. Gunduz A. Knight R. T. Schalk G. (2014). Spatial and temporal relationships of electrocorticographic alpha and gamma activity during auditory processing. *Neuroimage* 97 188–195. 10.1016/j.neuroimage.2014.04.045 24768933 PMC4065821

[B54] Power A. J. Foxe J. J. Forde E. J. Reilly R. B. Lalor E. C. (2012). At what time is the cocktail party? A late locus of selective attention to natural speech. *Eur. J. Neurosci.* 35 1497–1503. 10.1111/j.1460-9568.2012.08060.x 22462504

[B55] Pritchard W. S. Shappell S. A. Brandt M. E. (1991). “Psychophysiology of N200/N400: a review and classification scheme,” in *Advances in Psychophysiology* Vol. 4 eds Jennings FNMJ. R. Ackles cpsFNMP. K. Coles cpsFNMM. G. H. (London: Jessica Kingsley Publishers).

[B56] Reinhart R. M. G. Mathalon D. H. Roach B. J. Ford J. M. (2011). Relationships between pre-stimulus gamma power and subsequent P300 and reaction time breakdown in schizophrenia. *Int. J. Psychophysiol.* 79 16–24. 10.1016/j.ijpsycho.2010.08.009 20816708 PMC3033488

[B57] Ridderinkhof K. R. Ullsperger M. Crone E. A. Nieuwenhuis S. (2004). The role of the medial frontal cortex in cognitive control. *Science* 306 443–447. 10.1126/science.1100301 15486290

[B58] Ritter W. Simson R. Vaughan H. G. Friedman D. (1979). Brain event related to the making of a sensory discrimination. *Science* 203 1358–1361. 10.1126/science.424760 424760

[B59] Sauseng P. Klimesch W. Gruber W. R. Birbaumer N. (2008). Cross-frequency phase synchronization: a brain mechanism of memory matching and attention. *Neuroimage* 40 308–317. 10.1016/j.neuroimage.2007.11.032 18178105

[B60] Schroeder C. E. Lakatos P. (2009). Low-frequency neuronal oscillations as instruments of sensory selection. *Trends Neurosci.* 32 9–18. 10.1016/j.tins.2008.09.012 19012975 PMC2990947

[B61] Song J. Davey C. Poulsen C. Luu P. Turovets S. Anderson E. (2015). EEG source localization: sensor density and head surface coverage. *J. Neurosci. Methods* 256 9–21. 10.1016/j.jneumeth.2015.08.015 26300183

[B62] Stam C. J. Nolte G. Daffertshofer A. (2007). Phase lag index: assessment of functional connectivity from multi channel EEG and MEG with diminished bias from common sources. *Hum. Brain Mapp.* 28 1178–1193. 10.1002/hbm.20346 17266107 PMC6871367

[B63] Stam C. J. van Straaten E. C. (2012). The organization of physiological brain networks. *Clin. Neurophysiol.* 123 1067–1087. 10.1016/j.clinph.2012.01.011 22356937

[B64] Strauss A. Wostmann M. Obleser J. (2014). Cortical alpha oscillations as a tool for auditory selective inhibition. *Front. Hum. Neurosci* 8:350. 10.3389/fnhum.2014.00350 24904385 PMC4035601

[B65] Stropahl M. Bauer A. R. Debener S. Bleichner M. G. (2018). Source-Modeling Auditory Processes of EEG Data Using EEGLAB and Brainstorm. *Front. Neurosci.* 12:309. 10.3389/fnins.2018.00309 29867321 PMC5952032

[B66] Sutton S. Braren M. Zubin J. John E. R. (1965). Evoked-potential correlates of stimulus uncertainty. *Science* 150 1187–1188. 10.1126/science.150.3700.1187 5852977

[B67] Szalárdy O. Tóth B. Farkas D. Kovács A. Urbán G. Orosz G. (2018). The effects of attention and task-relevance on the processing of syntactic violations during listening to two concurrent speech streams. *Cogn. Affect. Behav. Neurosci.* 18 932–948. 10.3758/s13415-018-0614-4 29949114

[B68] Tadel F. Baillet S. Mosher J. C. Pantazis D. Leahy R. M. (2011). Brainstorm: a user-friendly application for MEG/EEG analysis. *Computat. Intell. Neurosci.* 2011:879716. 10.1155/2011/879716 21584256 PMC3090754

[B69] Tóth B. Farkas D. Urbán G. Szalárdy O. Orosz G. Hunyadi L. (2019). Attention and speech-processing related functional brain networks activated in a multi-speaker environment. *PLoS One* 14:e0212754. 10.1371/journal.pone.0212754 30818389 PMC6394951

[B70] Verleger R. (1988). A critique of the context updating hypothesis and an alternative interpretation of P3. *Behav. Brain Sci.* 11 343–356. 10.1017/S0140525X00058015

[B71] Watkins P. V. Barbour D. L. (2011). Level-tuned neurons in primary auditory cortex adapt differently to loud versus soft sounds. *Cereb. Cortex* 21 178–190. 10.1093/cercor/bhq079 20457692 PMC3000570

[B72] Wightman F. L. Kistler D. J. (2005). Informational masking of speech in children: effects of ipsilateral and contralateral distracters. *J. Acoust. Soc. Am.* 118 3164–3176. 10.1121/1.2082567 16334898 PMC2819474

[B73] Wild-Wall N. Falkenstein M. (2010). Age-dependent impairment of auditory processing under spatially focused and divided attention: an electrophysiological study. *Biol. Psychol.* 83 27–36. 10.1016/j.biopsycho.2009.09.011 19799963

[B74] Woldorff M. G. Hazlett C. J. Fichtenholtz H. M. Weissman D. H. Dale A. M. Song A. W. (2004). Functional parcellation of attentional control regions of the brain. *J. Cogn. Neurosci.* 16 149–165. 10.1162/089892904322755638 15006044

[B75] Womelsdorf T. Fries P. (2007). The role of neuronal synchronization in selective attention. *Curr. Opin. Neurobiol.* 17 154–160. 10.1016/j.conb.2007.02.002 17306527

[B76] Xia M. Wang J. He Y. (2013). BrainNet Viewer: a network visualization tool for human brain connectomics. *PLoS One* 8:e68910. 10.1371/journal.pone.0068910 23861951 PMC3701683

[B77] Zalesky A. Fornito A. Bullmore E. T. (2010). Network-based statistic: identifying differences in brain networks. *Neuroimage* 53 1197–1207. 10.1016/j.neuroimage.2010.06.041 20600983

[B78] Zhang C. Lu L. Wu X. Li L. (2014). Attentional modulation of the early cortical representation of speech signals in informational or energetic masking. *Brain. Lang.* 135 85–95. 10.1016/j.bandl.2014.06.002 24992572

