# Supplementary material for: Neuronal Correlates of Informational and Energetic Masking in the Human Brain in a Multi-Talker Situation
Source: Front Psychol. 2019 Apr 9;10:786. doi: 10.3389/fpsyg.2019.00786 (PMC6465330; doi:10.3389/fpsyg.2019.00786)
Supplement: Supplementary file 1 [file Data_Sheet_1.docx]

**Supplementary Material**

**Neuronal correlates of informational and energetic masking in the human brain in a multi-talker situation**

Orsolya Szalárdy^*^, Brigitta Tóth, Dávid Farkas, Erika György, István Winkler

*Correspondance: Orsolya Szalárdy: [szalardy.orsolya@ttk.mta.hu](mailto:szalardy.orsolya@ttk.mta.hu)

**1. Description of syntactic violations**

Syntactic violations were included in 18 of the 20 distractor streams (19-26 each M=20.5, SD=1.4). Because only 18 text segments with syntactic violations were recorded, two stimulus blocks out of the 20 did not contain these violations, always assigned to the moderately softer distractor stream condition. Note that our previous study showed that syntactic violations in the distractor speech stream did not elicit syntactic- or semantic-violation related ERP responses (Szalárdy et al. 2018). Syntactic violations (see Supplementary Table 1.) were divided into two subgroups: one in which the phrase structure of the sentence was violated (phrase structure violation) and the other in which inflection rules were violated (verb inflection violation). Phrase structure violation was realized by appending the object suffix to the subject word (M=8.3, SD=3.1, range: 2-14 per speech segment). Verb inflection violation was realized by employing a plural subject noun and in conjunction with a singular predicate verb or a singular subject noun together with a plural predicate verb (M=12.2, SD=3.0, range: 6-19); the subject could either precede or follow the predicate. The distance between the grammatically mismatching words (subject-predicate or object-predicate) never exceeded 4 syllables (maximum two words).

**Supplementary Table 1. Subtypes and examples for syntactic violations**

| Syntactic violation | Verb inflection violation with subject predicate agreement mismatch | | Phrase structure violation |
| --- | --- | --- | --- |
| Subtype | Plural subject noun  +  singular predicate verb | Singular subject noun  +  plural predicate verb | Appending the object suffix to the subject word |
| Example in Hungarian | Havonta átlagosan hatvan órán át **használja** az **emberek**… | A **videójáték-ipar** szintén **kihasználják**… | Később azonban az oktatási intézmények, majd végül több ezer **civilt** is csatlakozott… |
| Example in English with explanation | Monthly on average sixteen hour **use** (singular) the **people** (plural)… | The **videogame-industry** (singular) also **exploit** (plural)… | Later however the educational institutions and finally thousands of **civils** (object) joined. |

**2. Correlation between behavioral measures and significant FC results**

In order to determine whether the processes indexed by EEG FC are related to task performance, Pearson correlation was calculated between the average connectivity strength difference of the networks emerging from the two LOUDNESS contrasts (energetic and information masking) and the difference in various behavioral measures (d’, hit rate, false alarm rate, RT, and recognition performance) between the same two conditions. For compatibility with the networks emerging from the contrasts, behavioral measures were averaged across the moderately louder and slightly louder, and separately across the moderately softer and slightly softer conditions, before subtracting them from the corresponding measure in the equal loudness condition. FC strength difference was calculated as the average of the strength difference of the edges comprising the network. Thus correlations were calculated for the two masking effects (energetic and information masking), separately for the two possible effect directions (i.e., one for the subnetwork composed of edges showing stronger connectivity and another for that showing weaker connectivity in the given contrast), for each frequency band in which a significant network was found for the given contrast, and each behavioral measure. Potential effects were controlled by family-wise error calculated across the behavioral measures, separately for each contrast, effect direction, and frequency band.

**Supplementary Table 2.** Results of the Pearson correlation (r values) calculated between the average connectivity strength difference of the networks emerging from the two LOUDNESS contrasts (energetic and information masking) and the difference in various behavioral measures (d’, hit rate, false alarm rate, RT, and recognition performance) between the same two conditions. In all contrasts p > 0.05.

| **Frequency band** | **Contrast** | **Reaction time** | **d'** | **Hit rate** | **False alarm rate** | **Recognition performance** |
| --- | --- | --- | --- | --- | --- | --- |
| **Alpha** | Energetic masking | 0.0041 | 0.254 | 0.361 | 0.418 | 0.187 |
|  |  |  |  |  |  |  |
| **Gamma** | Energetic masking | 0.204 | -0.298 | -0.330 | -0.232 | 0.167 |
|  |  |  |  |  |  |  |
| **Theta** | Informational masking | -0.121 | -0.094 | 0.085 | 0.233 | 0.146 |
|  |  |  |  |  |  |  |
| **Beta** | Informational masking | -0.151 | -0.028 | 0.018 | 0.023 | 0.049 |
|  |  |  |  |  |  |  |


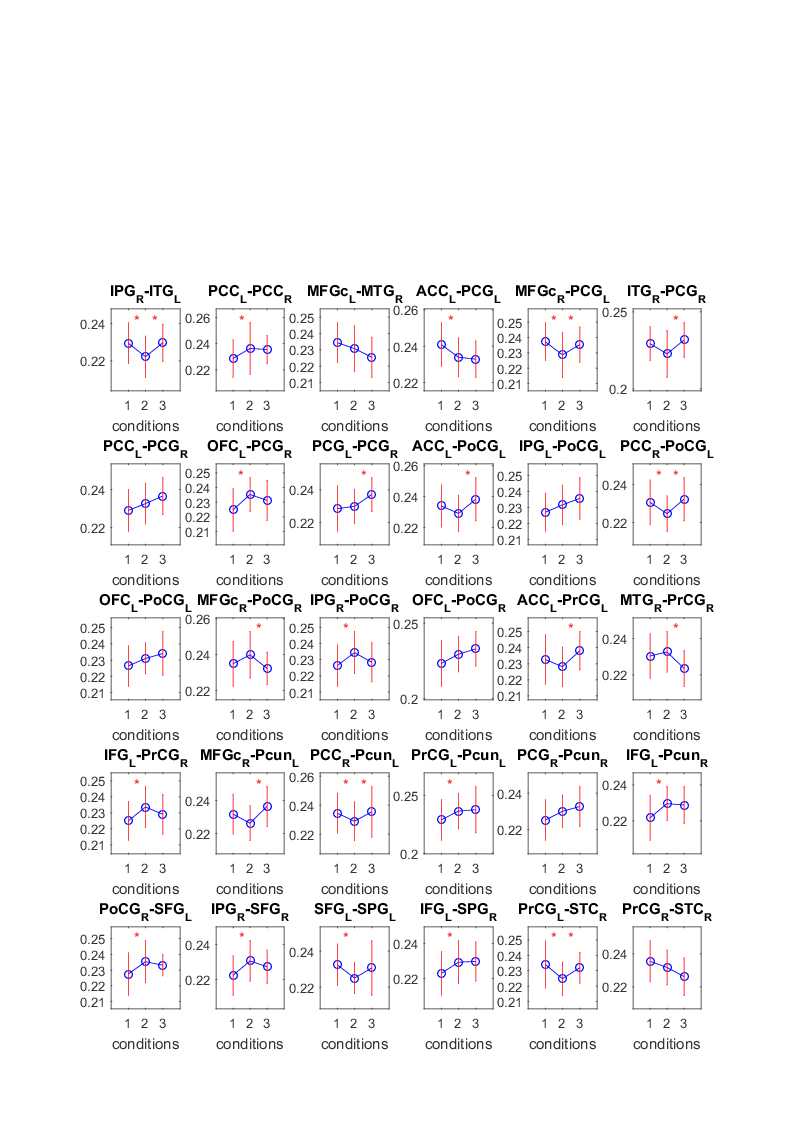
**Supplementary Figure 1.** Significant network of the post-hoc analysis testing monotonic FC strength behavior as a function of loudness difference. The direction of the connectivity strength did not showing monotonically increasing or decreasing trend except for four edges (ACC-PCG, PCG-PCG, PrCG-Pcun, IFG-SPG; ML: moderately louder distractor, SL: slightly louder distractor, E: equal loudness).

**Supplementary Table 3.** Summary of the number of connections for each node within the network that showed significant ENERGETIC MASKING or INFORMATIONAL MASKING effect (the two effect directions in separate sub-columns), separately for the four EEG bands (main columns). Each line represents a node (source brain area) which is further grouped by major lobes (Frontal, Cingular, Temporal, and Parietal).

|  | **ENERGETIC MASKING** | | | | **INFORMATIONAL MASKING** | | | |
| --- | --- | --- | --- | --- | --- | --- | --- | --- |
|  | **Low alpha** | | **Gamma** | | **Theta** | | **Beta** | |
|  | Equal | Louder | Equal | Louder | Equal | Softer | Equal | Softer |
|  | > | > | > | > | > | > | > | > |
|  | Louder | Equal | Louder | Equal | Softer | Equal | Softer | Equal |
| ***Frontal*** |  |  |  |  |  |  |  |  |
| PrCG | 2 |  |  |  | 3 | 2 |  | 4 |
| IFG | 4 |  | 2 | 1 | 1 | 3 | 1 |  |
| MFGr | 4 | 1 |  |  |  |  | 2 |  |
| MFGc | 1 |  |  |  |  |  |  | 4 |
| SFG | 1 | 3 |  |  | 1 | 2 |  |  |
| OFC | 4 |  |  |  | 2 | 3 | 2 | 1 |
| ***Temporal*** |  |  |  |  |  |  |  |  |
| STC | 1 |  | 1 |  | 1 |  |  |  |
| MTG | 4 |  | 1 |  | 2 | 2 | 1 |  |
| ITG | 1 | 1 |  |  |  |  |  | 2 |
| FFG | 7 |  |  |  | 1 |  |  |  |
| ***Cingular*** |  |  |  |  |  |  |  |  |
| PCC | 1 |  |  |  |  | 2 | 2 | 3 |
| ACC | 1 | 1 | 1 |  | 1 | 4 |  | 1 |
| ***Parietal*** |  |  |  |  |  |  |  |  |
| PoCG | 6 | 1 |  |  |  | 7 |  | 3 |
| PCG | 1 |  | 3 |  | 1 | 3 | 2 | 2 |
| SMG | 4 | 1 |  |  |  | 4 |  |  |
| IPG | 4 |  |  |  | 1 | 3 |  |  |
| SPG | 3 |  |  | 1 | 2 | 2 | 1 | 3 |
| Pcun | 4 |  |  |  |  | 3 |  |  |


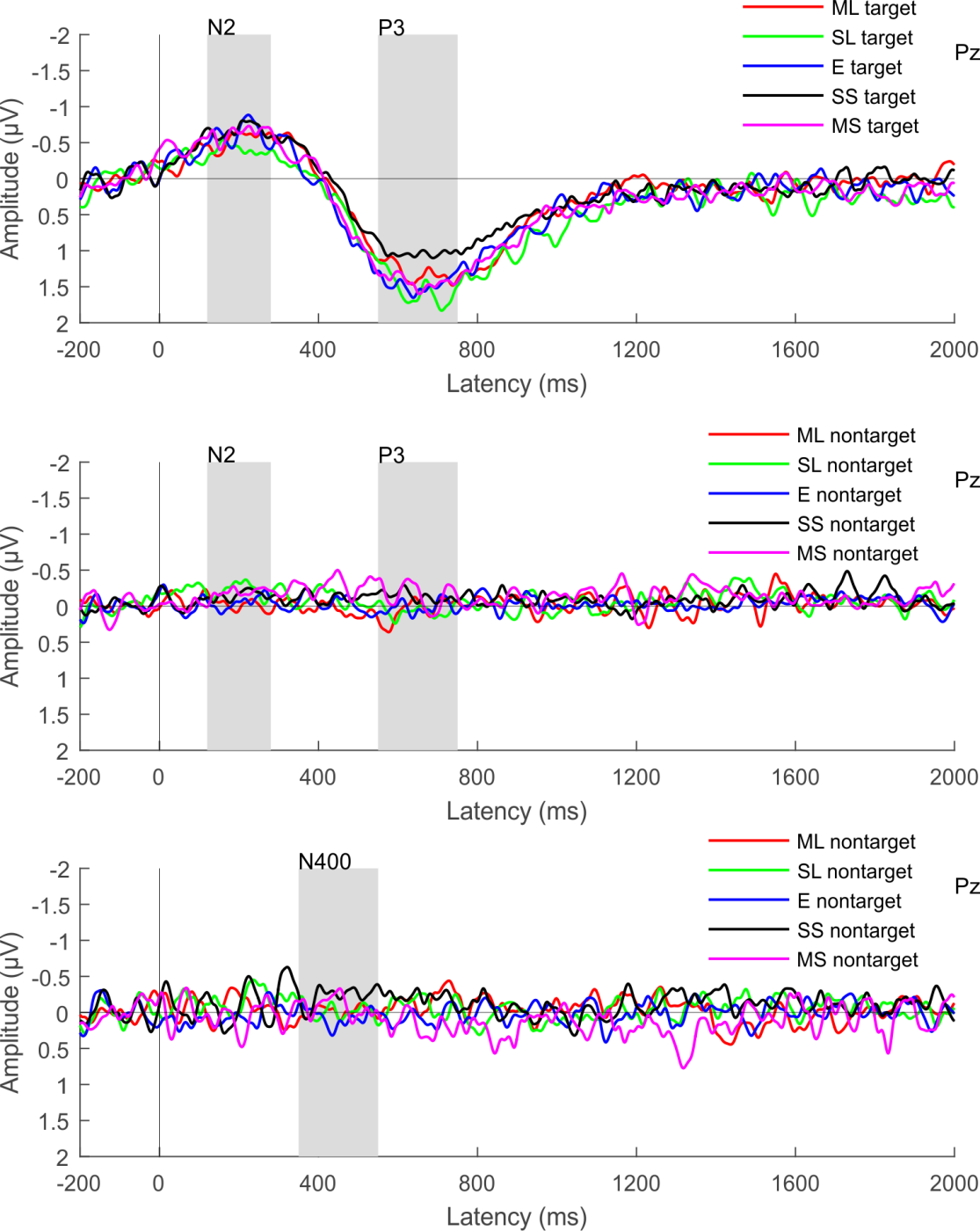


**Supplementary Figure 2.** Group-average (N=27) parietal (Pz) ERP responses elicited by non-target stream numerals (upper panel) and non-target stream syntactic violations (bottom panel). Color represents the different loudness conditions (ML= moderately louder, SL = slightly louder, E = equal loudness, MS = moderately softer, SS = slightly softer distractor); the N2b, P3b, and N400 latency ranges are shown by grey rectangles where applicable.
